# Announcing the Lancet Migration European Hub and the Lancet Regional Health-Europe Commission on climate change, migration, displacement and health

**DOI:** 10.1016/j.lanepe.2025.101532

**Published:** 2025-11-17

**Authors:** Bernadette N. Kumar, Anand S. Bhopal, Sylvia Garry, Rosemary James, Ozge Karadag, Miriam Orcutt, Shilpa Rao, Shervin Shahnavaz, Santino Severoni, Amirhossein Takian, Pooja Jha, Karl Blanchet

**Affiliations:** a Norwegian Institute of Public Health, Oslo, Norway; b Bergen Centre for Ethics and Priority Setting, Faculty of Medicine, University of Bergen, Bergen, Norway; c Health and Migration, World Health Organization, Geneva, Switzerland; d Geneva Centre of Humanitarian Studies, Faculty of Medicine, University of Geneva, Geneva, Switzerland; e Bahcesehir University (BAU), Faculty of Medicine, Istanbul, Turkey; f Centre for Psychiatry Research, Department of Clinical Neuroscience, Karolinska Institute, and Stockholm Health Care Services, Region Stockholm, Sweden; g Center of Excellence for Global Health (CEGH), Department of Global Health, School of Public Health, University of Medical Sciences (TUMS), Tehran, Iran; h The Lancet Regional Health – Europe, Munich, Germany

“Wicked problems”, described by Rittel and Weber^1^ 50 years ago, are issues that are difficult to define and hard to solve, owing to a combination of competing values, inherent uncertainties, and diverse interconnections. The nexus of climate change, migration, displacement and health exemplifies such a challenge. Climate change is a complex, politically sensitive, and ethically fraught challenge. It is deeply entangled with other global health challenges, such as infectious disease outbreaks, food insecurity, and health inequities, and increasingly shapes where, why, and how people relocate. ‘Migration’ is a broad concept which describes the movement of people away from their habitual place of residence regardless of legal status, reason, or length of stay, whereas ‘displacement’ refers more specifically to involuntary movement of people, particularly within borders. Population mobility, a multifaceted phenomenon, independently impacts health risks and resilience, depending on country of origin, conditions of displacement, reception, and integration.^2^^,^^3^ Understanding the intersections between climate change, migration and displacement in Europe underpins health system resilience, response and health improvements. These intersections underscore the urgent need for rigorous evidence to inform adapted policies and effective responses.

There is growing awareness of the importance of the climate change-health-migration-displacement nexus, including within the Intergovernmental Panel on Climate Change (IPCC)'s 6th Assessment report.^4^ Yet, climate change and migration health research are still largely conducted in silos, with different histories, approaches and disciplinary expertise. This disconnect calls for innovative, collaborative, and interdisciplinary research to help establish clear links between migration, displacement and climate change and untangle their direct and indirect health consequences in the near and long term.

Climate change is no longer a distant challenge. Europe is currently warming at approximately twice the global average resulting in cascading health impacts.^5^ The Lancet Countdown on Health and Climate Change shows that several health hazards—most notably heat-related mortality, climate-sensitive infectious diseases (e.g. West Nile virus, dengue, chikungunya), wild fires, flooding, and food insecurity driven by extended drought months—are rising in frequency, severity, and geographic reach, with Southern Europe particularly affected.^6^ Environmental risks are unevenly distributed. Ethnic minority and migrant communities, low-income populations, and those with insecure legal status facing disproportionately greater exposure to heatwaves and vector-borne disease.^6^ These inequities concur with the pattern of broader evidence: migrants and displaced populations often face disproportionate health risks and exposures, with poorer access to health care and worse health outcomes.^2^

Addressing knowledge gaps in the intersection of climate change, migration and displacement and health and identifying appropriate interventions are crucial to meet the needs of migrant and displaced populations in the European context. As data disaggregated by ethnicity, migration status, origin, and route are sparse, quantifying differential risk or long-term monitoring is difficult. Surveillance systems in many European countries seldom incorporate climate change indicators sensitive to migrant residence, legal status, or living conditions. Adaptation and resilience plans tend to focus on the general population, with limited attention to populations in informal settlements or those excluded by legal or administrative status.^7^ Strengthening research and monitoring, including longitudinal studies, to assess the impacts of climate shocks and long-term health consequences and integrating climate change-sensitive health indicators—such as those related to heat, air and water quality, and nutrition—into migration policies is imperative. Strengthening research and monitoring, including longitudinal studies, to assess the impacts of climate shocks and long-term health consequences and integrating climate change-sensitive health indicators into migration policies is imperative.

The Lancet Migration European Hub will lead this Commission which addresses the climate change-migration-health nexus, providing much needed evidence to inform policy development and responses on climate change, migration, displacement, and health across the WHO European region. As a science-led, multidisciplinary academic collegium, we will review the current global and European situation, identify urgent needs and gaps in research, policy, and practice, and produce original analyses and recommendations. Our work will include risk modelling to predict future migration flows, estimate health impacts, and support health-systems preparedness, using empirical evidence on the health-related pathways linking climate change events to migration and displacement.^8^ Understanding the links between climate change, migration, displacement and health and developing equitable responses will be incomplete without local stakeholder and migrant voices—participatory methods will be used throughout to incorporate these vital perspectives.

Our Commission will use a combination of quantitative and qualitative methods, complemented by case studies and vignettes (Fig. 1). Recognizing the inherently global phenomena under study, we will begin with the international context before focusing on the European region. At the global level, we will explore how a ‘Whole-of-Route approach’^9^ examining coordinated activities across countries of origin, transit, and destination—rather than focusing on arrival points alone—can support the health of migrants and displaced populations in the context of climate change. Within Europe, we will examine how climate change shapes migration patterns within, into, and out of the region and how these flows influence health and health systems, including unmet migrant health needs. We will also examine adaptation strategies and responses. This includes exploring whether existing European policies and practices on migration, health, and climate change adaptation are fit for purpose: do they sufficiently address the needs of migrants, and what innovative strategies could better address the climate change-health-migration nexus?

**Fig. 1:**
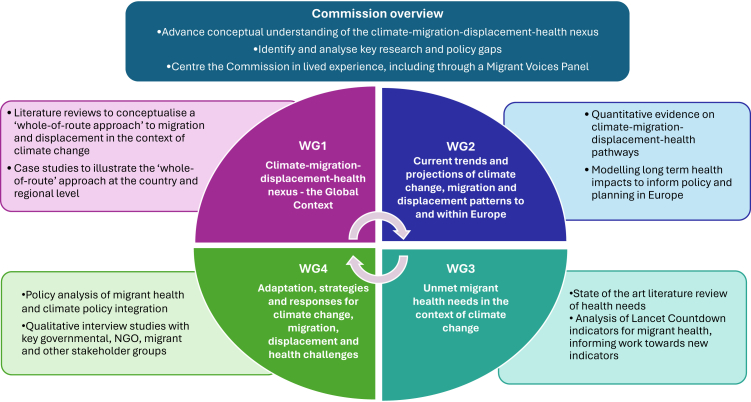
**Overview of the Commission on climate change, migration, displacement and health**. This figure displays the four working groups (inner circle), the key methodological approaches for each working group (outer boxes) and the overarching goals of the Commission (top).

Our work will advance knowledge and inform effective policy and practice. We will foster research that bridges and clarifies how climate change, migration and displacement interact, and elucidates their direct and indirect effects on health, both now and in the future. Addressing the climate-health-migration-displacement nexus requires cross-sectoral and cross-border cooperation. Ministries of health, environment, migration, labor, and housing must work together with local governments and civil society—including migrant-led organizations—for effective planning and implementation of policies. Our recommendations will highlight approaches for decision makers to join forces to predict and respond to the effects of climate change on population mobility and health, including through the development of indicators for the *Lancet Countdown on Health and Climate Change.*

Europe stands at a pivotal juncture where the principles of humanity, solidarity and universal health coverage are threatened. With climate change already causing measurable health impacts and migration and displacement patterns increasingly influenced by environmental stress, the need for integrated, rights-based public health planning is urgent. Failure to acknowledge and act on the nexus of migration, climate change, and health will perpetuate inequities, increase preventable morbidity and mortality, and undermine social cohesion. Emerging evidence highlights opportunities including inclusive health systems, developing climate change-sensitive and migration-sensitive policies, and fostering cross-sector partnerships that leave no one behind.^10^ These are not only moral imperatives; evidence-informed policies and actions are essential for health security, stability, sustainable development and the public good. By tackling the key challenges associated with climate change, health, migration and displacement, this Commission will advance the overarching objective of attaining the UN Sustainable Development Goals on good health and wellbeing (SDG 3), gender equality (SDG 5), reduced inequalities (SDG 10), more resilient cities and human settlements (SDG 11) and climate change and its impact (SDG 13).

## Contributors

BNK developed the draft manuscript, with substantial contributions from KB and ASB. All authors contributed to the development of the content, commented on drafts, and approved the final version of the manuscript.

## Declaration of interests

There are no competing interests.
